# Modulation of p53 binding to MDM2: computational studies reveal important roles of Tyr100

**DOI:** 10.1186/1471-2105-10-S15-S6

**Published:** 2009-12-03

**Authors:** Shubhra Ghosh Dastidar, David P Lane, Chandra S Verma

**Affiliations:** 1 Bioinformatics Institute (A-STAR), 30 Biopolis Street; #07-01 Matrix, Singapore 138671; 2 Institute of Molecular and Cell Biology (A-STAR), Proteos, 61 Biopolis Drive, Singapore 138673; 3 Department of Biological Sciences, National University of Singapore, 21 Lower Kent Ridge Road, Singapore 119077; 4 School of Biological Sciences, Nanyang Technological University, 50 Nanyang Avenue, Singapore 639798

## Abstract

**Background:**

The tumor suppressor protein p53 is regulated by the ubiquitin ligase MDM2 which down-regulates p53. In tumours with overexpressed MDM2, the p53-MDM2 interaction can be interrupted by a peptide or small molecule to stabilize p53 as a therapeutic strategy. Structural and biochemical/mutagenesis data show that p53 has 3 hydrophobic residues F19, W23 and L26 that embed into the ligand binding pocket of MDM2 which is highly plastic in nature and can modulate its size to accommodate a variety of ligands. This binding pocket is primarily dependent on the orientation of a particular residue, Y100. We have studied the role of the dynamics of Y100 in p53 recognition.

**Results:**

Molecular dynamics simulations show that the Y100 side chain can be in "open" or "closed" states with only the former enabling complex formation. When both p53 and MDM2 are in near native conformations, complex formation is rapid and is driven by the formation of a hydrogen bond between W23 of p53 and L54 of MDM2 or by the embedding of F19 of p53 into MDM2. The transition of Y100 from "closed" to "open" can increase the size of the binding site. Interconversions between these two states can be induced by the N-terminal region of MDM2 or by the conformations of the p53 peptides.

**Conclusion:**

Molecular dynamics simulations have revealed how the binding of p53 to MDM2 is modulated by the conformational mobility of Y100 which is the gatekeeper residue in MDM2. The mobility of this residue can be modulated by the conformations of p53 and the Nterminal lid region of MDM2.

## Background

**Figure 1:**
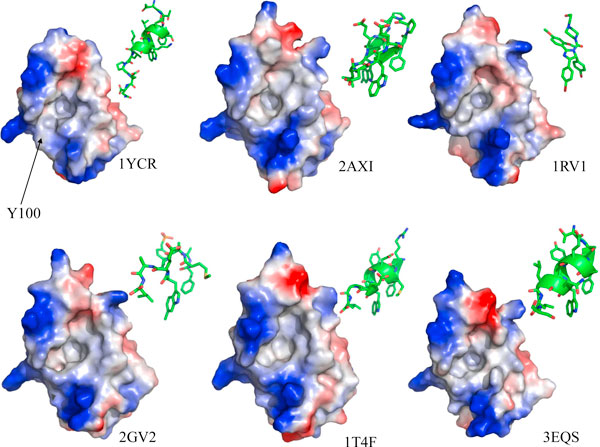
**MDM2 in surface representation, taken from various structures available in PDB, complexed with various ligands including WT p53 (**1YCR**), a β-hairpin peptide (**2AXI**), small molecule 'nutlin' (**1RV1**), an 8-mer p53 peptide analogue (**2GV2**), an optimized peptide (**1T4F**) and a 12 residue peptide**. PDB codes have been mentioned at the right bottom of each structure. The location of Y100 on the surface has been mentioned for the WT structure. The surface was colored according to the charges of the surface residues: positive (blue), negative (red) and neutral (white).

The tumor suppressing activity of the protein p53 is down-regulated by the ubiquitin ligase MDM2 which complexes p53 and targets it for degradation [1]. In normal cells the p53 protein, which also is a transcription factor active at the MDM2 promoter, is maintained at low levels through this negative feedback loop. In damaged cells the MDM2-p53 complex is destabilized through posttranslational modifications, e.g. phosphorylation. This disrupts the complex, frees p53 which then activates the repair or apoptotic pathways [1]. This has spawned several studies aimed at to the development of peptides/small molecules that can displace p53 from its complex with MDM2 [2-6]. The structural data on the MDM2-ligand complexes available in Protein Data Bank (PDB) show a wide structural variation amongst the several MDM2-inhibitor complexes. These reveal a highly plastic nature of the binding pocket of MDM2 (Figure 1). The significance of this plasticity in inhibitor design has been addressed in recent studies [7,8]. One residue that has been found to play a major role in modulating this plasticity through the size of the binding pocket is Y100 [7,9]. The orientation of the Y100 side chain controls the size of the binding pocket and also contributes to the stabilization of the ligands in the complex by enabling stable interactions. Both, the crystal structure (PDB code 1YCR) [10] and molecular dynamics (MD) simulations of the complex of MDM2 and a 13 residue fragment of the transactivation domain of p53 show that Y100 points away from the binding pocket and forms a hydrogen bond (HB) with either the backbones of E28 or N29 of wild type (WT) p53 peptide. This stabilizes an unstructured C-terminal region of the peptide that lies outside the binding pocket [7]. In addition, there are conformational states with small molecules/peptides bound where Y100 'points in' towards the binding pocket and stabilizes the complex with a different set of interactions [7]. Recently, it was reported from biochemical measurements that the P27S mutant of p53 has a higher affinity for MDM2 than wild type p53. Simulations revealed that in the complex, the peptide adopts an α-helical conformation at the C-terminus (which is unstructured in the WT p53) and a rearrangement of the network of interactions occurs. There is a dramatic change of the MDM2 surface which is caused by the reorientations of the L54 and Y100 side chains. The flip of the latter towards the binding pocket organizes a cozier fit of the ligand and stabilizes an HB with the L26 backbone, suggesting an induced fit mechanism of peptide binding [7].

**Figure 2:**
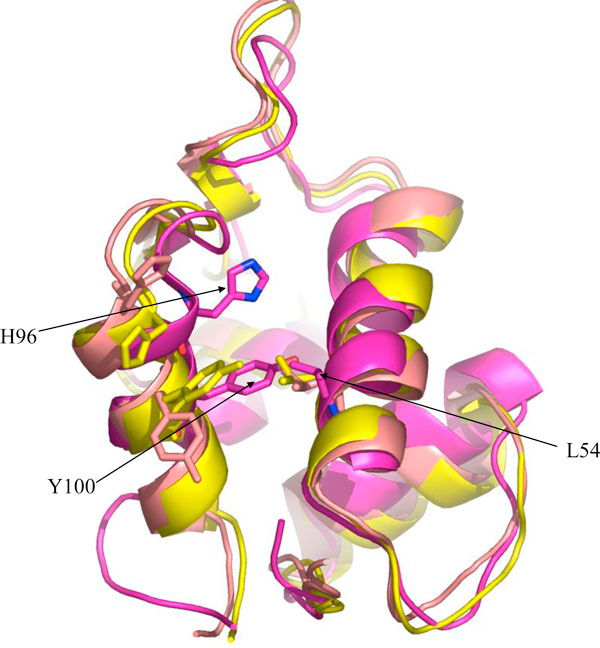
**Superimposed structures of 'open' (salmon red), 'closed' (yellow) and 'apo' (purple) states of MDM2**. The residues L54, H96 and Y100 have been shown in sticks for all conformations and the residue names have been labeled for the apo-state. The Y100 is deeply buried in the apo-state.

On the other hand, the apo state of MDM2 as evidenced by the ensemble of NMR structures (PDB code 1Z1M) [8] shows that Y100 sits in a deeply buried position (see Figure 2) which is remarkably different from that in its complexed state. This suggests a possible coupling of the dynamics of Y100 with the binding of p53. Interest has recently been focused on the differences between pre-organized binding/conformational selection (where for example the p53 peptide chooses a conformation of MDM2 from an ensemble, that is optimal to bind that peptide) or induced binding (for example the p53 peptide binds in some conformation to MDM2, and the complex then undergoes conformational rearrangements that maximizes the interactions) [11]. To investigate this we have carried out a series of molecular dynamics simulations of systems containing MDM2 and p53 in various states. The main focus is on investigating the changes that take place in p53 and in MDM2 as they approach each other. A somewhat similar investigation but with coarse graining simulations has recently been reported [12]. The study shows the conformational changes associated with the transition from uncomplexed (apo) state to the complexed states of MDM2 which leads to the diverse conformational states of MDM2.

We have used molecular dynamics simulations to examine three different conformations of MDM2: (i) 'Open' state of the conformation taken from the wild type (WT) complex (1YCR) with the Y100 side chain pointing away from the binding pocket; (ii) 'closed' state - the conformation of MDM2 taken from the complex with α-helical P27S mutant of p53 with the Y100 side chain pointing towards the binding pocket; (iii) The 'apo' state - the unliganded state of MDM2, obtained from the ensemble of NMR structures [8] where Y100 is in a deeply buried position (see Figure 2).

Other approaches to investigate such phenomena are Brownian Dynamics simulations [13], Replica Exchange methods [14-17] and the more recently developed accelerated molecular dynamics methods [18]. While the Brownian dynamics methods are well suited for studying protein-protein associations where proteins are depicted in a reduced representation [19], the replica exchange methods can be used to examine pathways of folding [16]; the accelerated molecular dynamics methods have been very successful in examining long time scale processes [18]. In this study, we are attempting to understand atomic level details of the process prior to the embedding of p53 into MDM2. Aspects of this process involve a coupling between folding and binding, at least of the p53 peptide [20]. We have chosen to run classical molecular dynamics simulations with several different starting conditions and as will be seen, these capture local folding/unfolding events through extensive surface rearrangements. A major factor that limits the usage of replica exchange methods here is that the current system is comprised of around 100 amino acids and replica exchange methods, particularly in explicit solvent, can only meaningfully (exhaustively) be applied to peptides that are up to 40 amino acids long [21-24] and hence are not suitable for the sort of extensive surface rearrangements that we sample here. Moreover, the MDM2 binding site is highly hydrophobic and there is the possibility that it will rapidly unfold in the REMD methods. For the same reason, the accelerated molecular dynamics method was not used.

## Results

We first outline briefly the currently accepted picture of the mechanism of interaction between p53 and MDM2 in their bound state. Crystallographic, biophysical and computational studies have traditionally shown that F19, W23 and L26 are the three critical residues of the transactivation domain (TA) of p53 which largely determine the stability of its complex with MDM2 [10,25]. The residues F19 to L25 form an α-helical segment which has a hydrophobic face that subtends the side chains of the three hydrophobic residues F19, W23 and L26 which get embedded in the binding pocket of MDM2. In addition to the hydrophobic interactions between these 3 residues and MDM2, the W23 side chain also makes an HB with the backbone of L54 of MDM2, and this is very critical for the stability of the complex [7,26]. More recently, it has been demonstrated that other parts of both p53 and MDM2 are involved in modulating these interactions [7,12,27]. One residue whose dynamics appear to potentiate this binding is Y100 of MDM2 which "gates" the conformation of the p53 peptide. This residue is conserved across species (see Supporing Information in [7]). If the p53 peptide is extended at its C-terminus (as seen in the crystal structure 1YCR), the side chain hydroxyl of Y100 is involved in hydrogen bond (HB) with the backbone of either E28 or N29 of the p53 peptide; however the p53 peptide can also adopt an α-helical conformation at its C-terminus and this is potentiated by the Y100 hydroxyl forming an HB with the backbone of L26 which provides a 'cozier' fit between the p53 peptide and MDM2 [7]. We set out to investigate the mechanism that governs the development of these interactions as p53 approaches MDM2; we are particularly interested in examining the modulation of the conformational activity of Y100. Recently, a study has investigated such a process using targeted simulations of the binding pathway under a coarse grained description [12]. In contrast, here we examine the process of binding, without directing the binding, and in an all-atom model with explicit solvation. The steady behavior of the root mean squared deviations (RMSD) of MDM2 in the various situations shows that the simulations are stable (see Figure S1 in Additional File 1).

### p53 and 'open' MDM2

**Figure 3:**
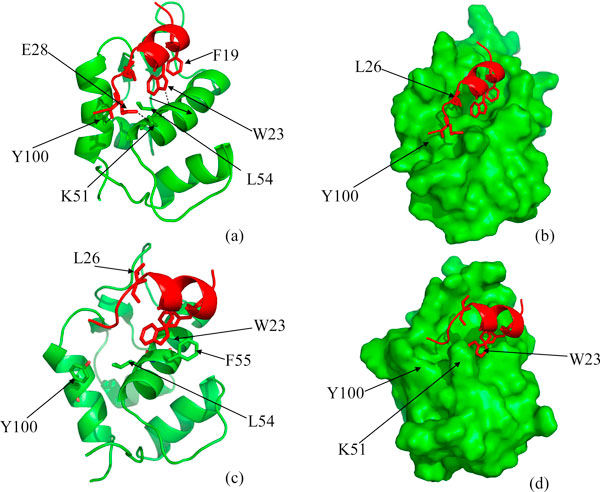
**(a),(b) Snapshots taken at the end of 10 ns simulation of Mo3A (See Table 1)**. MDM2 (green) has been shown in ribbon in 'a' and surface representations in 'b'; and p53 (red) has been shown in ribbon. The HB between residue pairs W23-L54, E28-Y100 and the polar interaction between the side chains of E28 and K51 have been shown with dashed (black) line in the picture 'a'. (c),(d) Snapshot taken at he end of Mc3A trajectory (See Table 1)

When p53 approaches the 'open' state of MDM2 from a distance of 3 Å, (trajectory: Mo3A, see Table 1), complex formation is rapid and the crystallographically observed state is reached with most of the interactions reported above reproduced. The W23-L54 HB forms within 300 ps. The Y100-E28 hbond takes somewhat longer (~2 ns) to form. Indeed, the complex is stabilized by 10 ns, which suggests that preorganization of the partners into 'reactive' conformations leads to rapid complex formation (Figure 3 and Additional File 2). This of course was enabled because we started with the crystal structure conformations (which are the preorganized conformations). This is in accord with the models proposed and observed in other simulations for protein-protein interactions [12,28]. Moreover, the enthalpy of binding close to what has been reported elsewhere using the crystal structure of the complex (ΔH~-56.6 kcal/mol here compared to -54.7 kcal/mol, [7]); the difference is expected due to the different starting states. Indeed it is heartening to see the similarity of the two values, suggestive of a real productive encounter between p53 and MDM2 starting 3 Å apart. Interestingly Y104, the residue spatially contiguous to Y100 undergoes side chain mobilities that are larger in magnitude to those in Y100 and yet are correlated with it (Figure S2 in Additional file 1).

**Table 1:** List of trajectories.

	Trajectory name	MDM2	p53	Length
1	Mo3A	open	RMSD 3 Å	10 ns
2	Mo6A	open	RMSD 6 Å	10 ns
3	Mc3AT1	closed	RMSD 3 Å	20 ns
4	Mc3AT2	closed	RMSD 3 Å	20 ns
5	Mc6A	Closed	RMSD 6 Å	40 ns
6	Mc6A7ns	simulation started from the snapshot taken at 7.5 ns of Mc6A		25 ns
7	M1n4A	Apo, model 1	RMSD 4 Å	20 ns
8	M1n6A	Apo, model 1	RMSD 6 Å	10 ns
9	M1n4AWT	Taken from M1n4A at the end of 20 ns	RMSD ~6 Å	5 ns
10	M1n	Apo, model 1	NIL	20 ns
11	M2n119	Apo, model 2 (Residue 1-119)	NIL	15 ns
12	M4n119	Apo, model 4 (Residue 1-119)	NIL	10 ns

The source of the coordinates of the structures has been reported in the text. The RMSD corresponds to the p53 backbone in the starting structures of the simulations, after superimposing the MDM2 of the on the crystal structure of the MDM2-p53 complex.

In contrast, when p53 approaches the "open" state of MDM2 from a separation of ~6 Å (Trajectory Mo6A), it manages to reach the surface of MDM2 in the vicinity of the binding pocket (Figure S1 in Additional File 1) within 0.5 ns. However the interactions are nonspecific and p53 never manages to embed into MDM2 completely. The fact that it reaches the surface originates in the long range electrostatic fields that will no doubt steer the two molecules [29]. The W23 side chain forms interactions with the side chain of F55 which appears to displace p53 to a position slightly away from its native (or crysatllographically observed) location. At the same time, Y100 flips in towards the binding pocket; this presumably happens to minimize the exposure to solvent of a very hydrophobic binding pocket of MDM2. Again the mobility of Y104 is correlated with that of Y100 (Figure S2 in Additional File 1).

### p53 and 'closed' MDM2

**Figure 4:**
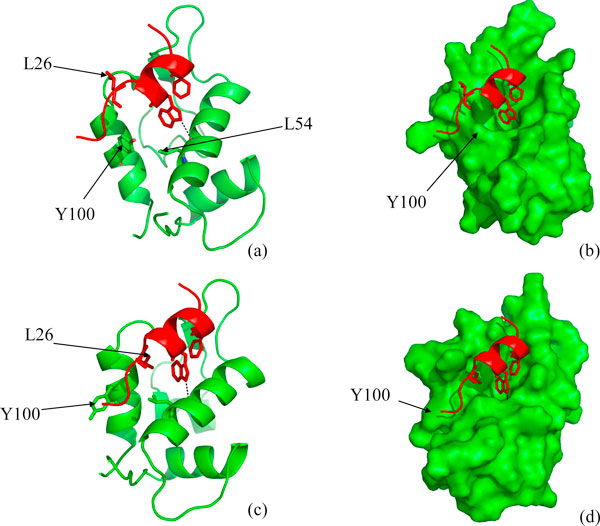
**(a),(b) Snapshot at the end of 20 ns simulation of Mc3AT1**. Y100 stays in a 'flipped-in' conformation and L26 of p53 (red) is out of the binding pocket, F19 and W23 fits into their crystallographically observed position and W23 and L54 forms HB; (c),(d) Snapshots at the end of 20 ns simulation of Mc3AT2. F19, W23 and L26 are fitted into the binding pocket of MDM2. Critical H-bond between W23 and L54 is marked with dashed line. Figures in the same row represent the same structure but differ in ribbon and surface representations of MDM2 (green).

We next examine the approach of p53 towards MDM2 when the latter is in its 'closed' state. F19 and W23 find their crystallographically observed positions (trajectory Mc3AT1, Table 1) within 0.5 ns, but L26 is obstructed by Y100 which points into the binding pocket (this being the 'closed' state of MDM2). The HB between the W23 side chain and the L54 backbone is transiently stable because the W23 is prevented from approaching sufficiently close to MDM2 (Figure 4 and Additional File 3). This is because of competition between the W23 side chain and the Y100 side chain for Hbonding with the backbone of L54 and also because the surface of MDM2, as defined by an inward-pointing Y100, occludes p53. Eventually L26 is "kicked out" by Y100 (See Movie in Additional File 3). In the trajectory Mc3AT1 Y100 is further forced to remain in its closed conformation by the extended C-terminus of the p53 peptide. To further probe the effect of the restraint imposed upon Y100, we modified the initial conformation of the C-terminus of p53. The backbone conformations of residues 27-28 were changed in a manner that released the spatial constraint upon Y100. Now we observe (trajectory Mc3AT2) that Y100 flips out after just a few nanoseconds and in turn enables p53 to nicely fit into the binding pocket of MDM2, in a conformation resembling that of the WT crystal structure (Figure 4 and Additional File 4); this clearly highlights the effect of the C-terminal region of p53 on modulating the gating of Y100 of MDM2. The mobility of Y104 is correlated strongly with that of Y100; interestingly, when no binding takes place, the magnitude of fluctuations of both side chains are similar, while upon binding events, the mobility of Y104 is correlated with that of Y100 but the magnitude is larger (Figure S2).

**Figure 5:**
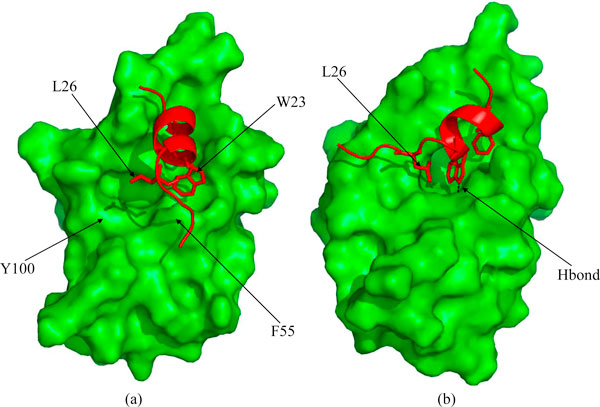
**(a) Snapshots taken at the end of 20 ns simulation of Mc6A**. W23 of p53 (red) is out of the binding pocket. (b) Snapshot taken at the end of 22 ns of the simulation of the trajectory Mc6A7ns, showing the crystallographically observed binding mode of the side chains F19, W23 and L26 and W23-L54 HB is formed.

When p53 was placed 6 Å away from the binding pocket of MDM2 (trajectory Mc6A), again, as seen in the "open" state described above, p53 reaches the surface of MDM2 in the vicnity of the binding pocket within 0.5 ns. In contrast to the "open" state, here Y100 flips out to fully open the binding pocket. Even as F19 attempts to dock, it provides an anchor that enables W23 and L26 to move towards their binding pockets. However, local collisions induce a rotation in the W23 side chain away from the orientation optimal for its binding. This results in p53 moving away from the surface of MDM2 and the W23 side chain adopts a stacking interaction with F55 of MDM2. (Figure 5 and Additional Files 5, 6, 7). We extracted a structure of the p53-MDM2 complex from this trajectory which whose conformation had the lowest deviation from the crystallographic complex (RMSD ~4 Å), and that had Y100 pointing "out"; this was subject to an MD simulation (Trajectory Mc6A7ns). This structure forms the crystallographically observed interactions in less than a nanosecond, including the W23-L54 HB (Figure 5b and movie Additional File 7). Again Y104 mobility is correlated with that of Y100 (Figure S2 in Additional File 1). Here we see some evidence of the presence of an intrinsic harmonicity with open-closed states of the Y100 appearing at a timescale of ~3-4 ns.

### p53 and 'apo' MDM2

**Figure 6:**
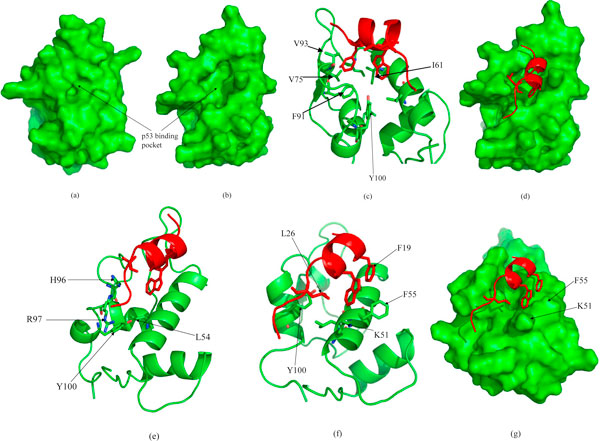
**(a) Surface representation of the Model 1 of the NMR ensemble (1Z1M), showing the absence of a well defined pocket**. This conformation has been used to run M1n4A after placing the p53. (b) MDM2 surface is showing an well defined pocket after simulation in presence of p53 (M1n4A), p53 has not been shown for clarity. (c) Hydrophobic Packing of F19 of p53 (red) in the binding pocket of MDM2 (green) at the end of 20 ns simulation (M1n4A) and (d) the complex same conformation with MDM2 in surface representation. Y100 is deeply buried, pointing towards the core of MDM2. (e) shows the snapshot from M1n4WT at the end of 5 ns simulation, which does not form the HB between W23 and L54; (f),(g) are the snapshot taken at the end of 10 ns simulation of the trajectory of M1n6A, shown in two different representations.

Thus far we have used the complexed state of p53-MDM2 determined crystallographically to seed the starting conformations of our various investigations. Given that the structure of MDM2 is available in its uncomplexed or "apo" state from NMR [8], we decided to use this as our starting state for MDM2. In all the 24 conformations of MDM2 available, the Tyr100 points inwards; this is expected as this minimizes the exposure of the very hydrophobic binding pocket of MDM2 and also enables the HB between the backbone of Leu54 and the side chain hydroxyl of Tyr100. When p53 was placed near the binding pocket of MDM2 (trajectory M1n4A), surprisingly p53 loses its helicity. This appears to be due to the fact that the side chains of MDM2 in this NMR-derived state are not optimally located to interact with p53 (which the crystallographic state does). However, as the system explores the conformational states, we soon observe that MDM2 interacts with W23 and F19 of p53 and its binding pocket is induced to open (Figure 6). Although this does not lead to the crystallographically observed state of the complex, W23 and F19 do embed in the binding pocket which leads to stabilization of the hydrophobic pocket of MDM2. F19 gets packed by several hydrophobic residues including I61, V75, F91, V93 and F86 (Figure 6c). The interesting observation is that although the orientation of the side chains of F19 and W23 change to a non native conformation, we do not see dissociation of the complex. It appears that at the initial stage of complex formation, helical conformations of p53 can initiate complex formation that can be seeded by an initial insertion of F19 into the hydrophobic pocket. This in turn is complemented by some expansion of the overall pocket of MDM2 that enables W23 to get embedded (See Movies in Additional Files 8).

To examine these later stages of binding we take the last (at a time of 20 ns) snapshot of the above trajectory and replace p53 (which has been somewhat distorted conformationally from its native bound conformation) by p53 taken from the crystal structure. We find that despite p53 being in its optimal conformation, in these simulations Y100 (which points in toward the binding pocket) does not enable p53 to embed and indeed pushes it away. In addition, the side chains of H96 and R97 obstruct the C-terminus of p53. Once again, Y100 is constrained by the presence of the C-terminal end of the TA of p53 (as we saw earlier in the case of p53 at 3 Å from the closed state) and cannot flip out to create space for W23 to embed. This indicates that prior to the binding of p53, the opening of the hydrophobic pocket of MDM2 by transition of Y100 to its 'open' conformation appears to be a key step to facilitate this binding. The interesting feature is that the mobility of Y104 is now dependant upon the position of the lid. The movies in the additional files 9, 10, 11 show that that once the lid opens, Y100 goes into an open state but Y104 is still constrained by the lid and does not open.

## Discussion

The TA of p53 is known to be largely intrinsically disordered with some parts of it adopting a helical conformation upon binding to MDM2. This brings three side chains (F19, W23 and L26) of p53 to be displayed on the same face of the helix thus enabling them to embed into a hydrophobic binding pocket of MDM2. Experimental data show that the unliganded state or the apo-state of MDM2 (derived using NMR, [8]) is quite different from its complexed state (derived using crystallography, [10]). What is not understood well is the process of binding of p53 and MDM2 to each other, mediated by these conformational states: is it pre-organization prior to binding or is it reorganization after binding (or induced fit). To test these, we have carried out a series of MD simulations that mimic the approach of p53 to MDM2. We avoided the simulation of folding/unfolding transitions of p53 during the simulations [20] and assumed that when p53 is very close to MDM2, stereo-chemical constraints would demand that the helical conformation of p53 be dominant in its interactions. We have mostly focused on the plasticity of MDM2 and the dynamics of Y100. The structural data shows that the plasticity of the binding pocket of MDM2 is mainly determined by the orientation of Y100 (Figures 1, 2), with spatially contiguous Y104 correlated with Y100 in mobility. When p53 is in its native (or crystallographically observed) state and MDM2 is "open", binding occurs with only small local reorganizations as side chains reorient minimally to maximize interactions. The size of the binding site, as determined from the solvent accessible surface area (data not shown) varies from ~2250 Å^2 ^to ~2450 Å^2 ^as the MDM2 transits from a relatively closed apo state to the p53 bound state. The general fluctuations of MDM2 (Figure S3 in Additional File 1) are conserved in pattern across the various simulations but the magnitudes vary. This is understandable because the peptide and MDM2 modulate each other and this will certainly change depending on their relative orientations, with the largest mobility witnessed in the presence of the lid (but absence of peptide). The one outlying feature is the high mobility of the 30-45 region when p53 is actually binding to MDM2 in approximately the crystallographically observed mode. While this region is distal to the p53 binding side, nevertheless the fact that it is high compared to the equilibrium dynamics [7] suggests that real equilibrium has not been achieved (this is ok for the purposes of our current study which only aims to examine the processes that occur as p53 approaches MDM2). When p53 is distant from MDM2, Y100 can assume both "in" and "out" states, with only the "out" state enabling binding. The binding process requires an initial encounter complex that is driven by nonspecific forces where electrostatic steering plays a major role. This is then accompanied by either the embedding of Phe19 or W23, that act as anchors across which the other two residues can be embedded. Both these residues, W23 and L26 require that Y100 is in an "out" conformation. This would enable the sidechain of W23 to make an HB with the backbone of L54 (which otherwise makes an HB with the hydroxyl of Y100). When Y100 is "in", it also occludes L26 from embedding. At the same time, when Y100 is "in", the C-terminal region of the TA of p53 also plays a critical role in modulating the dynamics of Y100 by preventing its transition to "open" states (at least in the timescales of these simulations).

**Figure 7:**
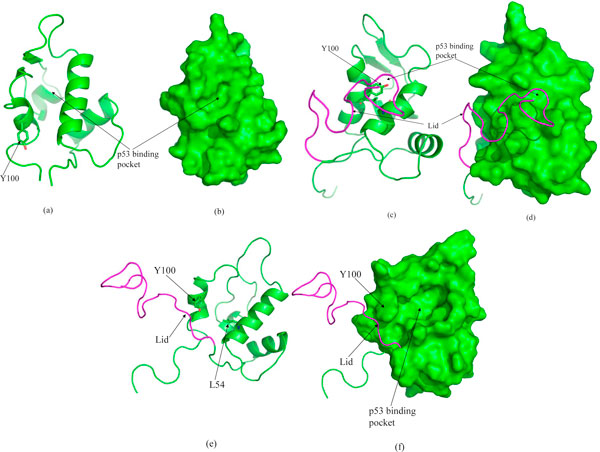
**(a),(b) Snapshot taken at the end of 20 ns simulation of 'apo' state only (Trajectory M1n)**. Y100 has flipped out. (c),(d) Snapshot taken at the end of 15 ns simulation of the 'apo' state of MDM2 containing the lid covering the MDM2 surface (residue 1-119, Trajectory M1n119). Y100 remains in the deeply buried situation and no well defined binding pocket has been observed. (e), (f) Snapshot taken at the end of 15 ns simulation of the 'apo' state of MDM2 containing the lid but not in contact with the MDM2 surface. Y100 flips out and well defined binding pocket has been observed. Figures in each row correspond to same structure but in two different representations: ribbon and surface. Surfaces have been shown for residues 25-109 only. The lid residues (1-24) have been shown in purple color.

The NMR ensemble shows that the "apo" states of MDM2 have Y100 "in" [8]. This is to minimize the exposed hydrophobic surface of the binding pocket. What is interesting is that these "apo" states, derived from NMR, had their N-terminal 24 residues (the lid) removed prior to simulations (in presence of p53 in the system). If the lid is included in the simulations (in absence of p53), there are two scenarios (Figure 7 and Additional Files 9, 10, 11). In some of the structures, the lid is localized over the binding site and pushes against Y100 and even during simulations, Y100 remains "in". However, there are some structures where the lid is localized away from the binding pocket. Simulations of these structures show that the restraint of the lid on Y100 is rapidly released and Y100 assumes an "out" conformation (Figure 7 and Additional Files 9, 10, 11). The same happens of the lid is deleted and then simulated in the absence of p53. This once again highlights the importance of the restraints that the local environment places upon the gating of Y100. Y100 in turn modulates the size of the binding pocket and also controls the local polarity by releasing the L54 backbone as a potential hydrogen bonding partner (for W23). Thus this suggests that a re-organization or induction to "open" and "closed" from "apo" states may be induced by the lid (or other factors). These in turn can be modulated by the presence of p53 and can either directly bind p53 (open Y100) or when p53 can induce an open state from a closed state by the conformational dynamics of the C-terminal end of the TA domain. This starts assuming significance biologically for several reasons: (a) the control of the size/polarity of the binding pocket by Y100 can affect the binding of p53 (and related proteins such as p73) and small molecules. We have shown how the orientation of Y100 modulates the thermodynamics of the peptidic inhibitors [7], a feature also demonstrated in other computational studies [30,31]; (b) the increasing importance of the MDM2 lid region, which is strictly conserved in mammals, in modulating the binding of p53 [32,33]; it is possible that by gating the dynamics of Y100, the lid region may influence the kinetics of binding of p53. The lid is known to compete only very weakly with peptidic inhibitors of MDM2 [34]. However to our knowledge, there is no published data available on the kinetics of peptide binding as modulated by the lid region in MDM2. The work of Worall *et al*. [33] has shown that the lid dynamics mostly modulate the ligase activity of the MDM2 which involves interactions of the lid with a region away from the p53 TA binding N-terminal region. It is clear that there must be a correlation between lid dynamics and substrate access as has been also found in several other systems as lactate dehydrogenase, lipases, adenylate kinases [35] (c) these questions assume greater significance with recent observations pointing to the increasingly complex interactions between MDM2 and p53 [30,31,36,37]. While our findings of the role of Y100 in modulating the equilibrium dynamics of the complex are in agreement with the findings of others [30] the added insights brought about by our studies, i.e., the modulation of the dynamics of the peptide and protein even before the binding event has taken place, is particularly relevant to understanding the on-rates of different peptides and perhaps may provide insights into the development of peptidic therapeutics whose rates of binding may be tunable.

## Conclusion

Molecular dynamics simulations have revealed how the binding of p53 to MDM2 is modulated by the conformational mobility of Y100 which is the gatekeeper residue in MDM2. They also reveal how the mobility of this residue itself can be modulated by the conformations of p53 and by the conformations of the Nterminal lid region of MDM2.

## Methods

The 'open' state of MDM2 (residues 25-109) was obtained from the crystal structure of the MDM2-p53 complex (RCSB entry 1YCR, resolved at 2.6 Å) [10]. The 'closed' state of MDM2 (residues 25-109) was obtained from our previous work [7]. The structure of the 'apo' state was chosen as Model 1 of the NMR ensemble [8] and although this structure consists of residues 1-119, we chose only residues 25-109 to be consistent with the other structures of MDM2 that have been used in the current and previous work. In order to examine the dynamics of the approach of p53 and MDM2 towards each other, we carry out all-atom simulations with MDM2 and p53 separated by varying distances (Table 1). We have also carried out simulations of MDM2 alone with its N-terminal 1-24 residues included as well. The CHARMM22 force field [38] was used to represent the systems. Each system was solvated using TIP3P water molecules and neutralized using counter-ions as required. After brief energy minimizations, each system was heated to 300 K followed by equilibration under constant pressure and temperature. Then molecular dynamics (MD) simulations at constant temperature and volume were carried out on each system for periods varying between 5-40 ns, yielding a total sampling time of ~200 ns. SHAKE [39] was applied to freeze the vibration of bonds involving hydrogen atoms, thus enabling a 2fs integration time step to be used. The Berendsen thermostat [40] was applied to keep the temperature constant. Simulations were carried out using the CHARMM package. Enthalpy was calculated using the MMGBSA approximations, with the GBSW [41,42] implicit solvent model. Figures were prepared using PYMOL [43] and movies were generated using VMD [44].

## Competing interests

The authors declare that they have no competing interests.

## Authors' contributions

SGD and CSV conceived the idea, SGD carried out the calculations and analysis. SGD and CSV wrote the manuscript. DPL discussed, advised and edited the manuscript.

## Note

Other papers from the meeting have been published as part of *BMC Genomics *Volume 10 Supplement 3, 2009: Eighth International Conference on Bioinformatics (InCoB2009): Computational Biology, available online at http://www.biomedcentral.com/1471-2164/10?issue=S3.

## Supplementary Material

Additional file 1 PDF format contains RMSD plots, average fluctuations, information of sampling of key side chain dihedrals etc. Click here for file

Additional file 2 Movie file of Trajectory Mo3A, red ribbon represents p53 and green ribbon represents MDM2. Few important side chains have been shown in 'sticks'. H-bonds appear as dashed lines. Click here for file

Additional file 3 Movie file of Trajectory Mc3AT1, coloring scheme is same as M1, The surface representation of this movie is available at http://web.bii.a-star.edu.sg/~shubhragd/paper_movies Click here for file

Additional file 4 Movie file, Trajectory Mc3AT2, shows the conformational transition of Y100 and p53 binding. The same movie but having MDM2 in 'surface' representation is available at http://web.bii.a-star.edu.sg/~shubhragd/paper_movies/ Click here for file

Additional file 5 Movie file, Trajectory Mc6A. Click here for file

Additional file 6 Movie file, Trajectory Mc6A, side view Click here for file

Additional file 7 Movie file, Trajectory M1n4A7ns Click here for file

Additional file 8 Movie file, Trajectory M1n4A Click here for file

Additional file 9 Movie file, Trajectory M1n, showing dynamics of Model 1 of NMR ensemble after deletion of residues outside the range of 25-109. The conformational change of Y100 side chains is observed. Click here for file

Additional file 10 Movie file, Trajectory M2n119. Shows the dynamics of MDM2 when the lid is close to the binding pocket. The Y100 has been shown in purple color for clarity. The Movie shows that Y100 stays in a deeply buried state when the lid is present. Click here for file

Additional file 11 Movie file, Trajectory M4n119. Shows the dynamics of MDM2 when the lid is away from the binding pocket. Y100 flips and points away from the binding pocket. Click here for file
