# Supplementary material for: Modulation of p53 binding to MDM2: computational studies reveal important roles of Tyr100
Source: BMC Bioinformatics. 2009 Dec 3;10(Suppl 15):S6. doi: 10.1186/1471-2105-10-S15-S6 (PMC2788357; doi:10.1186/1471-2105-10-S15-S6)
Supplement: Additional file 1 — PDF format contains RMSD plots, average fluctuations, information of sampling of key side chain dihedrals etc. [file 1471-2105-10-S15-S6-S1.pdf]

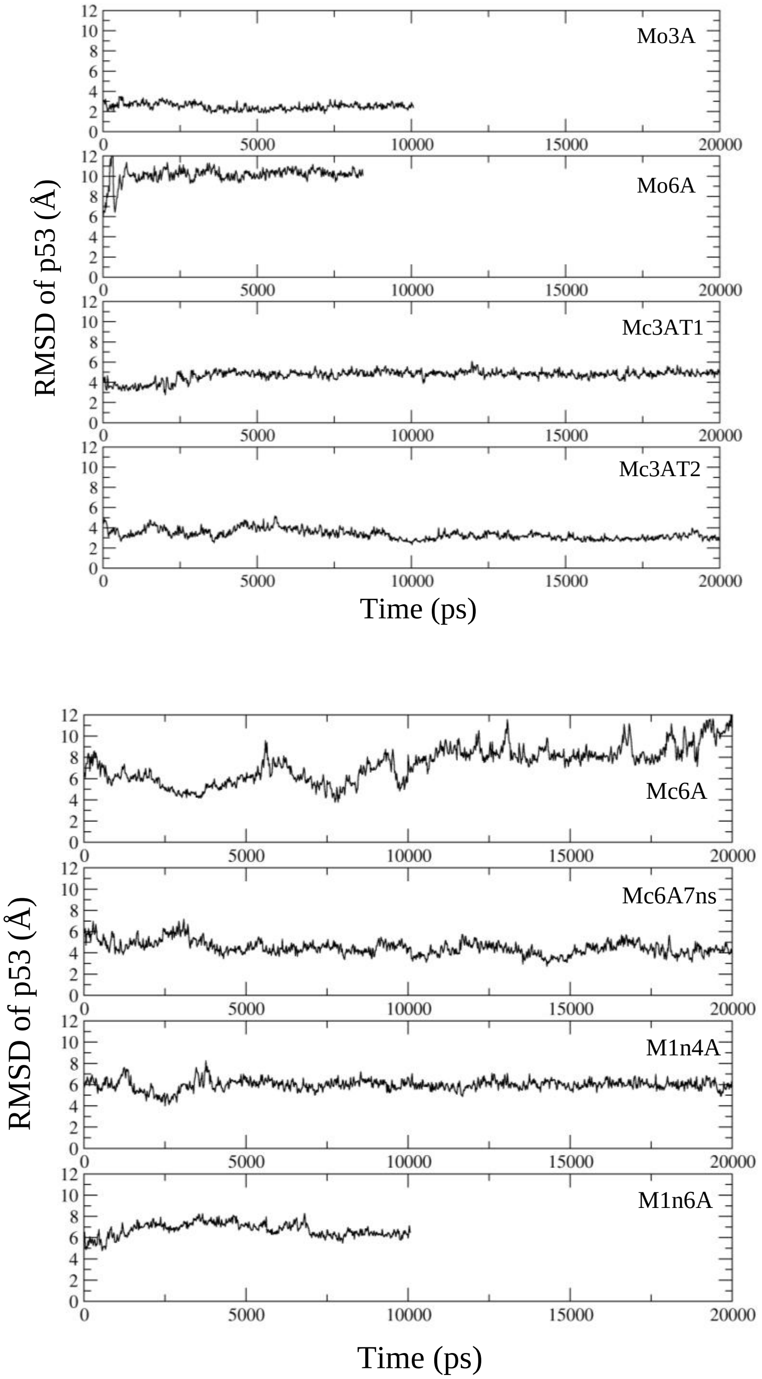

Figure S1(a): RMSD of all heady atoms p53 along the different trajectories. RMSDs have been calculated with respect to the crystal structure (1YCR), after superimposing the MDM2 backbones.

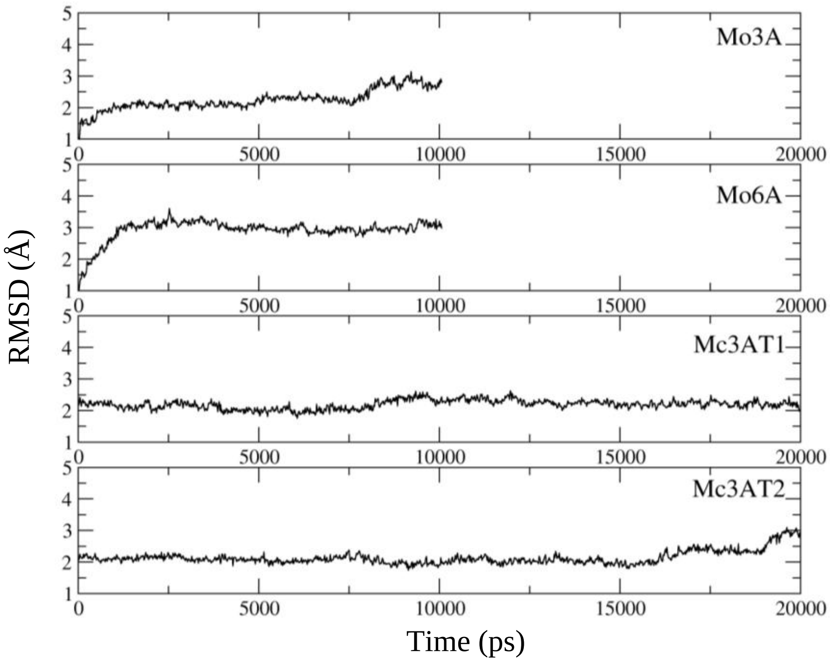

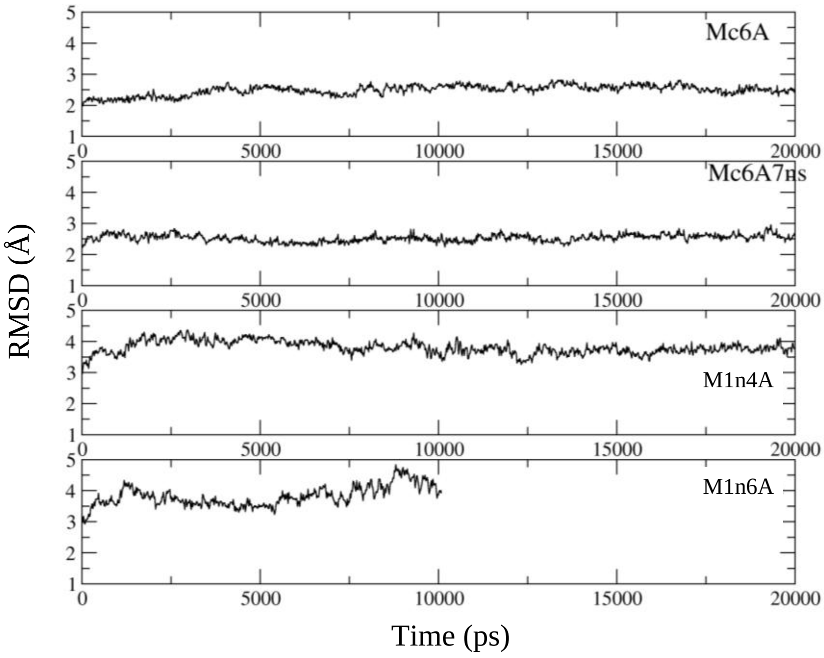

Figure S1(b): RMSD of all heady atoms MDM2 along the different trajectories. The MDM2 in the crystal structure of the MDM2-p53 complex (1YCR) was taken as reference.

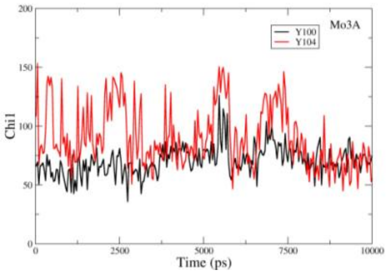
Mo3A
Y100
Y104
Chi1
Time (ps)

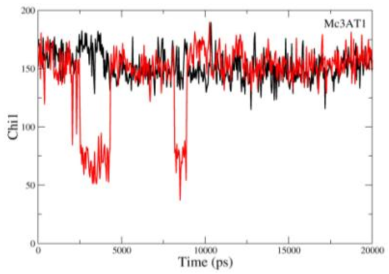
Mc3AT1
Chi1
Time (ps)

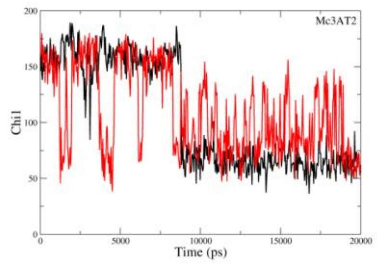
Mc3AT2
Chi1
Time (ps)

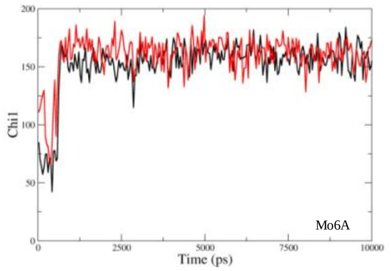
Mo6A
Chi1
Time (ps)

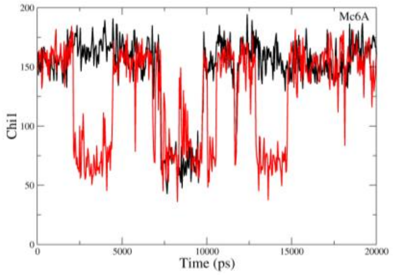
Mc6A
Chi1
Time (ps)

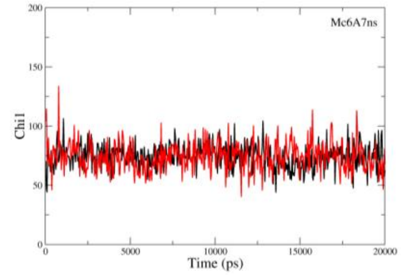
Mc6A7ns
Chi1
Time (ps)

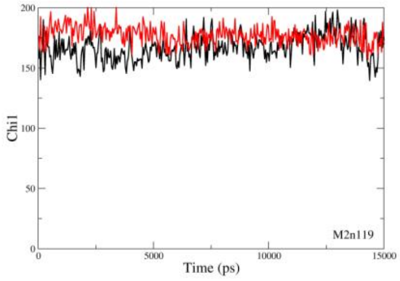
M2n119
Chi1
Time (ps)

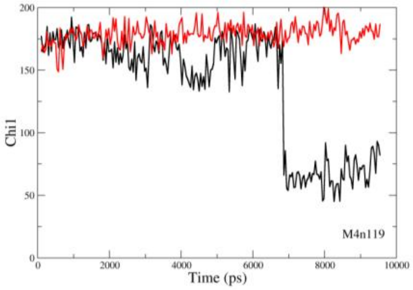
M4n119
Chi1
Time (ps)

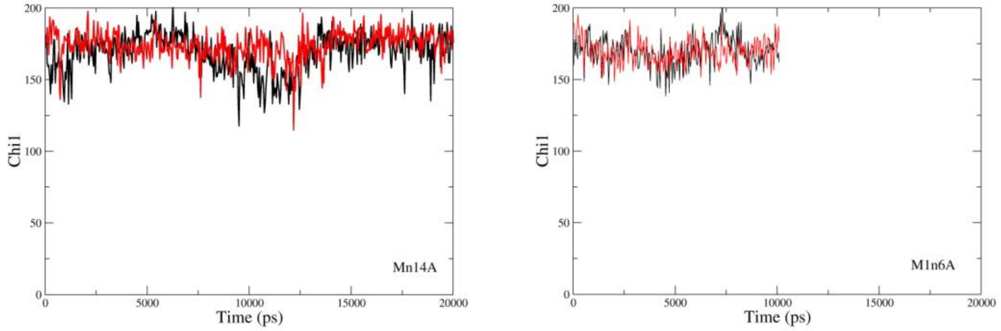

Figure S2: The Chi1 ($\chi1$, in degree) dihedral angle of the side chain of Y100 (black) and Y104 (red) as a function of time. The value of the angle around 60-80 degrees indicates an open conformation and that is around 150-180 degrees denotes closed conformation. Sharp transition between the states have been open-close have been observed. High correlation between the Chi1 angles of Y100 and Y104 has been observed as most of the time they follow same conformation. The MDM2 with closed (M2n119) conformations maintains Y100 in closed conformation too, whereas with open-lid (M4n119) the Y100 flips to open up around 7ns of the simulation.

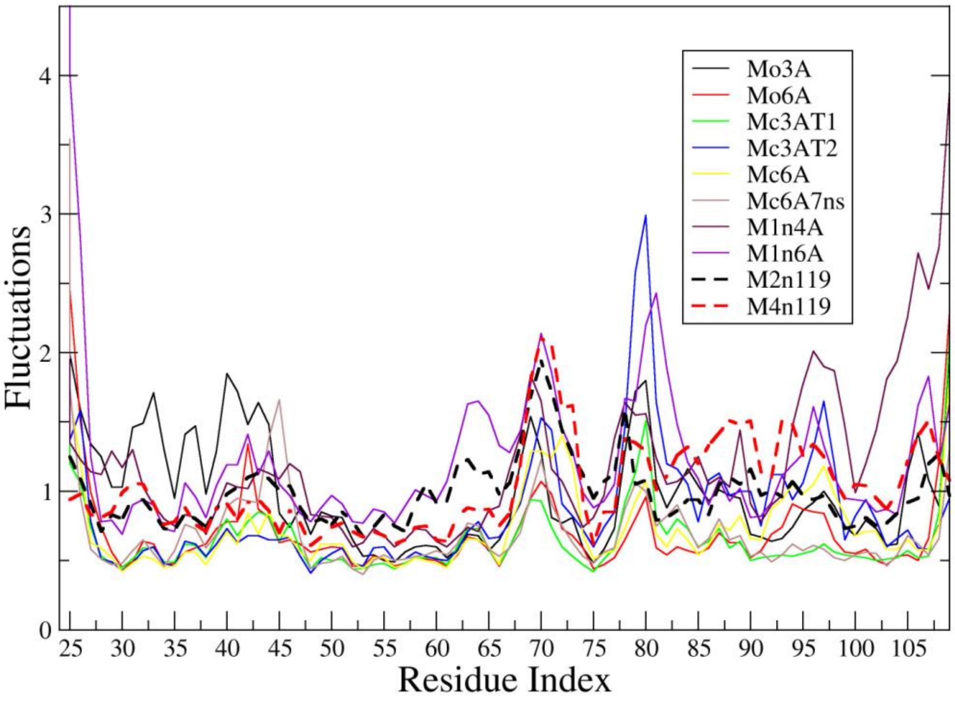

Figure S3: Averaged fluctuations of Cα atoms of MDM2. For the trajectories containing the lid residues, only the range 25-109 have been shown.

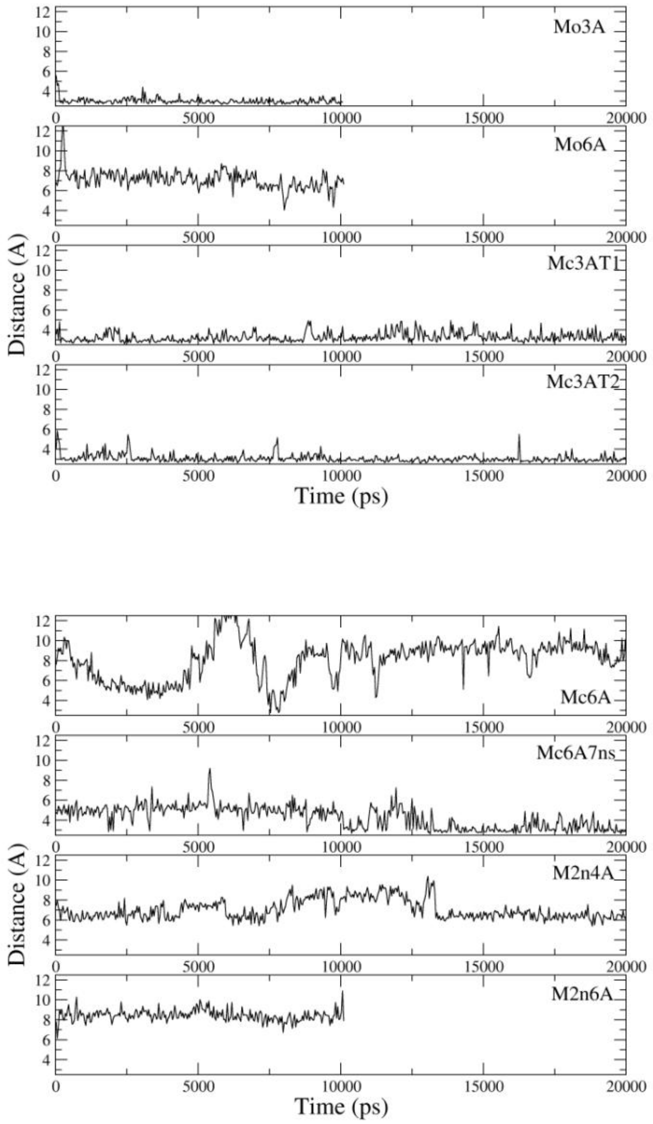

Figure S4: Time series of the distance between the backbone ‘O’ atom of L54 or MDM2 and side chain ‘N’ atom of W23 or p53 as a measure of complex formation. The Hbond between these two atoms (N-H······O) are crucial for the stability of the native complex (see Figures in the main text).
